# Sleep Supports Selective Retention of Associative Memories Based on Relevance for Future Utilization

**DOI:** 10.1371/journal.pone.0043426

**Published:** 2012-08-16

**Authors:** Eelco V. van Dongen, Jan-Willem Thielen, Atsuko Takashima, Markus Barth, Guillén Fernández

**Affiliations:** 1 Radboud University Nijmegen, Donders Institute for Brain, Cognition and Behaviour, Nijmegen, The Netherlands; 2 Radboud University Medical Centre, Department for Cognitive Neuroscience, Nijmegen, The Netherlands; 3 Radboud University Nijmegen, Behavioral Science Institute, Nijmegen, The Netherlands; University of Tasmania, Australia

## Abstract

An outstanding question is whether memory consolidation occurs passively or involves active processes that selectively stabilize memories based on future utility. Here, we differentially modulated the expected future relevance of two sets of picture-location associations after learning. Participants first studied two sets of picture-location associations. After a baseline memory test, they were instructed that only one set of associations would be retested after a 14-hour delay. For half of the participants, this test-retest delay contained a night of sleep; for the other half the delay included a normal working day. At retest, participants were re-instructed and against their expectations tested on both sets of associations. Our results show that post-learning instruction about subsequent relevance selectively improves memory retention for specific associative memories. This effect was sleep-dependent; it was present only in the group of subjects for which the test-retest delay contained sleep. Moreover, time spent asleep for participants in this sleep group correlated with retention of relevant but not irrelevant associations; participants who slept longer forgot fewer associations from the relevant category. In contrast, participants that did not sleep forgot more relevant than irrelevant associations across the test-retest delay. In summary, our results indicate that it is possible to modulate the retention of selected memories after learning with simple verbal instructions on their future relevance. The finding that this effect depends on sleep demonstrates this state’s active role in memory consolidation and may have utility for educational settings.

## Introduction

Recently encoded memories are initially unstable but become stabilized over time through a process known as consolidation [1], [2], [3]. Sleep appears to play an important role in this process [4], [5]. In the past, it was believed that sleep-related memory benefits were largely due to lack of interference from external sensory input [6], [7]. However, it is now becoming clear that sleep is actively influencing our memories [7], [8], [9], [10].

Based on recent evidence, sleep is not only actively, but also selectively participating in the stabilization of our memories. For instance, sleep has been shown to be involved in the preferential consolidation of memory for emotional objects within neutral scenes [11]. Compared to memory for neutral objects within neutral scenes, and memory for the neutral background itself, memory for emotional objects within neutral scenes was found to be selectively facilitated by sleep [11]. Further support for emotion as a driving force for sleep-dependent memory consolidation came from two recent studies that demonstrated that sleep stabilizes emotional over neutral memories [12], [13].

In addition, sleep appears to selectively boost the retention of information that is explicitly cued to be remembered during encoding [8], [14], suggesting that intentions during memory acquisition might also play a role in the selectivity of consolidation processes during sleep. Furthermore, a selective benefit for episodic details (as compared to item memory) was found in two recent experiments, indicating that not only emotional salience and encoding intentions, but also the type of memory might differentiate stabilization of particular information during sleep [15], [16].

An adaptive and active consolidation mechanism can provide a clear functional advantage: it allows for selective retention of experiences based on relevance for future utilization, thus maximizing the usefulness of long-term memory while limiting the strain on its capacity. As such, sleep-dependent consolidation might act as a filter mechanism, stabilizing and strengthening those memories that might be of importance later on. This notion is supported by data from a recent word pair association study in humans [17]. There, the knowledge that recently encoded word pairs would be tested in the future improved memory retention only for the group of participants that slept during the delay between learning and test [17]. However, this effect might have been based on a general facilitation of the consolidation process, rather than a re-activation of a particular memory, since the instructions on the future relevance of the word pairs were given in an all-or-none fashion. Consequently, the retention comparison was between experimental groups that knew or did not know that the learned word-pairs would be retested during the second experimental session. The between-subjects design used in that study therefore precludes any judgment on whether the observed effects were specific to the memories tested. A similar experimental manipulation, specific to parts of the learned material (e.g. in a within-subject design) would give more insight on this issue.

Additionally, the relevance of particular memories might be directly related to the benefits that are expected to be associated with their long-term retention. In line with this idea, a recent study has shown that sleep-dependent consolidation of procedural memories can be facilitated by reward expectancy induced after learning [18]. Specifically, in this study the expectation of reward for performing well on a specific motor sequence after a delay enhanced offline consolidation of this motor sequence when the test-retest delay contained sleep [18]. These results suggest that it is possible to modulate the offline stabilization of specific procedural memories with post-learning instructions. However, whether it is possible to selectively modulate the retention of specific declarative memories in this way is as yet unclear.

**Figure 1:**
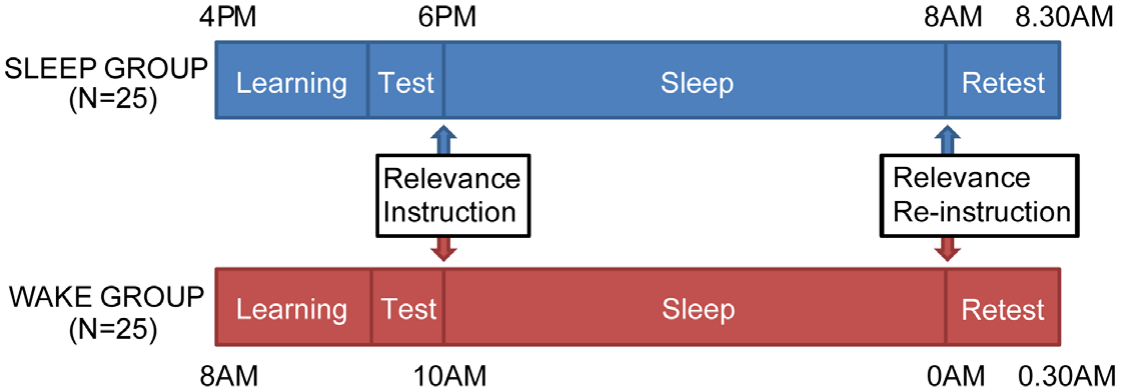
Schematic overview of the experimental paradigm. Participants learned two sets of picture-location associations and were tested on their memory for these stimuli at baseline (“TEST”). Subsequently, the relevance instruction was given. After a 14 hr delay, containing either normal daytime behavior (WAKE group) or a night of sleep (SLEEP group), participants were re-instructed and underwent a second memory test (“RETEST”) for all associations.

**Figure 2:**
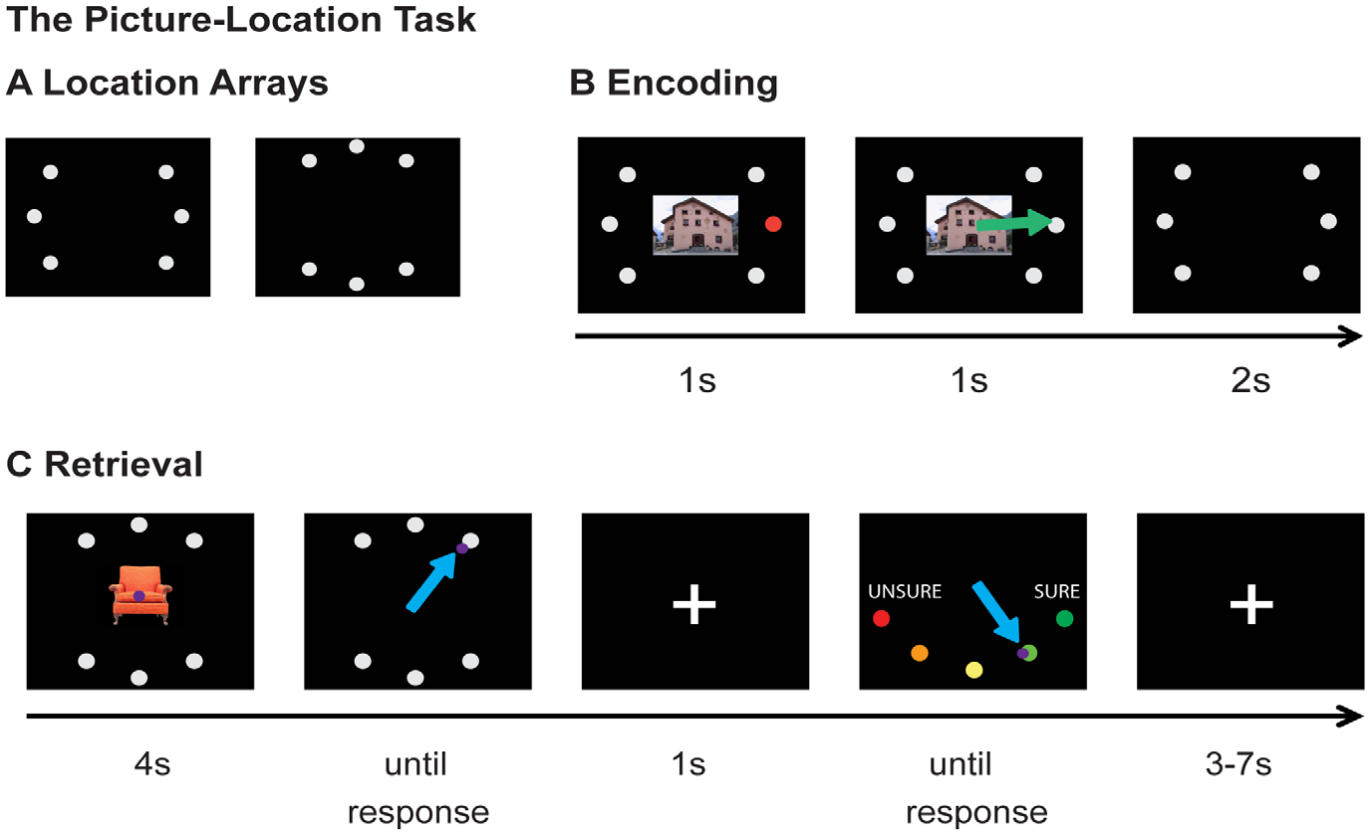
The Picture Location Task. *A. Location Arrays*. Picture categories were assigned one of the two location arrays in a counterbalanced manner across participants. Each location was associated with 10 pictures from the same category. ***B. Encoding.*** Participants passively watched as each picture was shown and placed at one of the six locations on the screen. The red dot informs the participant about the correct location of the current picture stimulus. The green arrow (not visible to the participant) indicates the automatic movement of the picture stimulus to this location. ***C. Retrieval.*** Participants used a joystick to indicate the correct location for each picture on the screen. The picture stayed on screen for 4s, but subjects were allowed to respond as soon as the picture appeared. After each response, participants provided a confidence rating. The purple dot represents the joystick cursor. Blue arrows indicate the joystick movements of the participant.

Our goal in this study was therefore to investigate if post-learning instructions on the future relevance of declarative memories can selectively modulate their subsequent consolidation. For this purpose, we modulated the expected future relevance of two sets of picture-location associations after learning (Figure 1). Sixty participants learned 120 picture-location associations with picture stimuli belonging to one of two categories (buildings or furniture; 60 pictures per category). Learning consisted of three encoding/retrieval cycles (see Figure 2). In every cycle, all pictures were first shown on their correct location (encoding phase) after which the participant actively retrieved the associations by attempting to select this correct location for each picture (retrieval phase). Presentation of pictures of both categories was intermixed and in random order for each cycle. At the end of the learning period, baseline performance for all associations was assessed during the retrieval phase of the third cycle. Participants were then instructed that this initial baseline TEST would be followed by an identical RETEST after a 14 hour delay. However, they were told that this retest would only include one category of pictures (the “RELEVANT category”), and not the other (the “IRRELEVANT category”). The assignment of building and furniture pictures to the two relevance categories was counterbalanced across subjects. In line with previous work [18], a monetary bonus was promised for each correctly recalled association from the RELEVANT category to further increase the behavioral relevance of retention. For half of the participants, learning occurred in the morning, with RETEST at night, and no sleep in between (the “WAKE group”). For the other half, learning occurred in the late afternoon, with RETEST in the morning, and the delay included a normal night of sleep (the “SLEEP group”). Participants’ sleep and activity during the delay was monitored for both groups using wrist-mounted actigraphy (Actigraph, Pensacola, USA). After the delay, participants were re-instructed against their expectations that they would be retested on both relevant and irrelevant categories, and would receive a monetary bonus for each correctly recalled association, regardless of category.

Based on previous research [17], [18], we hypothesized that retention of associations belonging to the RELEVANT category would be better than retention of pairings from the IRRELEVANT category. However, as we predicted that this effect would depend on sleep-related processes, we expected this relevance benefit to be specific to the SLEEP group and thus absent in the WAKE group.

## Results

### Performance

**Figure 3:**
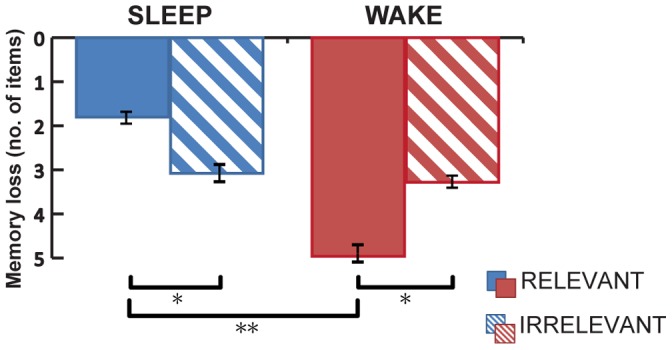
Relevance modulated retention of picture-locations associations. A 3-way interaction was observed between SESSION, RELEVANCE and GROUP. Performance is shown here as the amount of forgetting between TEST and RETEST (the effect of SESSION, “memory loss”, Y-axis). Analysis showed that sleep decreased memory loss of relevant compared to irrelevant associations, whereas daytime wake showed an opposite effect. Error bars denote the standard error of the mean. Brackets indicate significant differences in post-hoc comparisons. * = p<0.05, ** = p<0.005.

In our analyses, we used the hit rate ( =  number of correctly recalled picture-location associations) at the TEST and RETEST as the primary outcome variable. Fifty participants were included in our data-analyses; 10 participants (4 from the WAKE group, 6 from the SLEEP group) were excluded from the analysis because they reported doubts or had suspicions about the relevance instruction during the debriefing. A 3-way Repeated Measures ANOVA including the factors SESSION (TEST vs RETEST), GROUP (WAKE vs SLEEP) and RELEVANCE (RELEVANT vs IRRELEVANT) showed a significant 3-way interaction (F = 13.19, p = 0.001) and a main effect of SESSION (F = 54.67, p<0.001). No main effect of GROUP, RELEVANCE or any significant 2-way interaction between the 3 factors was found (all p>0.05). Post-hoc comparison using 2-tailed paired T-tests revealed that performance decreased significantly from TEST to RETEST in both SLEEP and WAKE groups (WAKE: relevant (−5.0±0.9; t = 5.59, p<0.001), irrelevant (−3.3±0.6; t = 5.11, p<0.001)) & (SLEEP: relevant (−1.8±0.2; t = 3.70, p = 0.001), irrelevant (−3.1±0.1; t = 4.49, p<0.001)), see Table 1 and Figure 3), in line with previous results using similar paradigms [19]–[21]. We then conducted a post-hoc analysis of the 3-way interaction using a 2-way Repeated Measures analyses (with the factors RELEVANCE and SESSION) for the SLEEP and WAKE group separately. This revealed that in the SLEEP group, relevant associations were retained better than associations from the irrelevant category (RELEVANT-IRRELEVANT difference (Δ)± standard error of the mean difference  = 1.3±0.6; F = 4.49, p = 0.045). In contrast, in the WAKE group, retention of relevant associations was worse than retention of irrelevant associations (Δ = −1.7±0.6; F = 9.44, p = 0.005). Specifically, within the SLEEP group, 15 out of 25 participants showed better retention of relevant compared to irrelevant associations (4 showed no difference; 6 showed the opposite effect). In comparison, 18 out of 25 participants from the WAKE group showed better retention of irrelevant compared to relevant associations (3 showed no difference; 4 showed the opposite effect). A comparison of test-retest change scores using a two-way ANOVA with the factors RELEVANCE and GROUP showed a significant RELEVANCE x GROUP interaction (F = 13.19, p = 0.001) but no main effects of RELEVANCE or GROUP (both p>0.05). Post-hoc analyses showed that the SLEEP group showed better retention of relevant associations than the WAKE group (Independent Samples T-test on the test-retest difference: Δ = 3.2±1.0; t = 3.12, p = 0.003). However, no significant difference in retention between SLEEP and WAKE groups was observed for the irrelevant category (Δ = 0.2±0.9; t = 0.21, p = 0.830), suggesting that sleep benefited only associations of future relevance.

**Table 1:** Mean performance for the WAKE and SLEEP group.

Group	Condition	Test	Retest	Difference
Wake	Relevant	50.8(±0.4)	45.8(±0.5)	−5.0(±0.1)
	Irrelevant	48.9(±0.5)	45.6(±0.5)	−3.3(±0.1)
Sleep	Relevant	54.2(±0.3)	52.4(±0.4)	−1.8(±0.2)
	Irrelevant	54.3(±0.3)	51.2(±0.4)	−3.1(±0.1)

*Performance is listed as the number of correct responses (maximum  = 60) with the Standard Error of the Mean in brackets.*

### Between-group Comparison of Baseline Performance

A comparison of the number of correct responses between the SLEEP and WAKE group at TEST showed no significant difference in overall performance (p>0.05), suggesting that time-of-day effects did not affect baseline performance. However, separate analyses of TEST performance for the RELEVANT and IRRELEVANT associations in both groups showed that participants in the SLEEP group performed significantly better than participants in the WAKE group in the IRRELEVANT condition at TEST (IRRELEVANT (ΔSLEEP-WAKE, mean ± SEM): 5.4±2.5, p = 0.04; RELEVANT: 3.4±2.2, p = 0.132). The future relevance of the two categories of stimuli was not known to participants when they performed the retrieval task at TEST, and therefore could not have affected performance. Nevertheless, such a baseline difference between groups could possibly introduce a confound to our analysis. We therefore repeated the analyses of the previous section while excluding the 2 participants from the WAKE group that performed most poorly. This abolished all significant differences in performance between groups (all p>0.05). Using this approach, all effects reported in the previous section remained significant. A 3-way Repeated Measures ANOVA including the factors SESSION (TEST vs RETEST), GROUP (WAKE vs SLEEP) and RELEVANCE (RELEVANT vs IRRELEVANT) again showed a significant 3-way interaction (F = 14.75, p<0.001) and a main effect of SESSION (F = 57.00, p<0.001). Moreover, a two-way ANOVA on the test-retest difference with the factors RELEVANCE and GROUP demonstrated a significant RELEVANCE x GROUP interaction (F = 11.94, p = 0.002) but no main effects of RELEVANCE and GROUP (both p>0.05), similar to the results observed when including the complete WAKE group. Post-hoc analyses confirmed that retention continued to be worse for RELEVANT than IRRELEVANT associations in the WAKE group (Δ = −1.8±0.5, F = 9.44, p = 0.002). Together, these findings show that equating performance between experimental groups at TEST did not change our results in a meaningful way. We therefore found no support for the idea that the effects we observe here are due to differences in baseline performance between groups.

### Confidence and Reaction Times

We investigated whether relevance and/or sleep modulated confidence ratings and reaction times recorded during the experiment. A 3-way Repeated Measures ANOVA on the participants’ confidence ratings for each trial with the factors SESSION (TEST vs RETEST), GROUP (WAKE vs SLEEP) and RELEVANCE (RELEVANT vs IRRELEVANT) showed only a significant main effect of SESSION (F = 45.4, p = <0.001) and no significant interactions. Reaction times for each trial were subjected to the same analytic design and provided comparable results (only a main effect of SESSION: F = 96.0, p<0.001). Post-hoc analyses showed that confidence ratings decreased across the delay, whereas reaction times became longer. However, in contrast with the performance changes reported in the main text, this happened uniformly across both groups of participants and similarly for both relevant and irrelevant associations.

### Sleep Duration and Performance

Finally, we analyzed the time participants in the SLEEP group spent asleep during the 14 hour delay using the actigraph recordings. A positive correlation was found between time spent asleep and the retention of relevant associations; participants who slept longer forgot fewer associations from the RELEVANT category (r = 0.41, p = 0.043). No significant correlations were found between sleep time and either overall retention or retention of irrelevant associations (overall retention: r = 0.14, p = 0.52; irrelevant retention: r = −0.09, p = 0.679). A direct comparison of Z-transformed correlation coefficients showed that the correlation between sleep time and retention for the RELEVANT category was greater than that between sleep time and retention for the IRRELEVANT category (Z = 2.50, p = 0.012).

## Discussion

We found a sleep-specific benefit of future relevance for memory retention. Our data show that post-learning instruction with regard to the future relevance of specific picture-location associations can selectively improve memory retention when followed by sleep. Interestingly, an opposing effect of post-learning instruction was found when the test-retest delay contained no sleep. These findings are similar to results from a study by Diekelmann and colleagues [22], in which reactivation of recent memories was actively prompted during sleep and wake. There, opposing consequences of such reactivation were observed along the lines of the effects reported here. We can only speculate on the origin of these effects. First, some evidence suggests that sleep can particularly benefit memories that are explicitly cued for remembering, as compared to those implicitly encoded or instructed to be forgotten [4], [8], [14]. Furthermore, consolidation seems to be sensitive to the emotional load of the memories that are encoded. Sleep appears to preferentially stabilize emotional over neutral memories [11]–[13]. For these reasons, it has been suggested by Saletin and Walker that sleep-dependent memory processing includes a selection mechanism that determines both retention and forgetting of items based on salience cues present during wake [10]. The relevance instruction that was given after learning in our study might have triggered this mechanism and thus facilitated the retention of the relevant associations over those classified as irrelevant. The increased salience of the relevant memory traces following the instruction might have facilitated offline reactivation of these traces and thus could have contributed to greater stabilization of the relevant associations across subsequent sleep [23]. Conversely, salience cues that are not followed by sleep but instead by normal daytime behaviour might have made the relevant associations more susceptible to interference from ongoing cognitive processes [24]. This could be one reason for the detrimental effects of relevance on retention across a wake delay period observed here.

Alternatively, it is possible that specific rehearsal of relevant associations following the instruction in the beginning of the delay period contributed to our findings. General rehearsal of the relevant associations apparently did not benefit retention, as the WAKE group (having the largest opportunity for rehearsal) showed decreased retention of these associations compared to the SLEEP group. Nevertheless, one could argue that a short period of rehearsal of relevant associations following learning could have led to a larger number of weakened relevant compared to weakened irrelevant associations for participants in the WAKE group, with the opposite effect occurring in the SLEEP group, along the lines of the findings reported by Diekelmann and colleagues [22].

Finally, sleep did not appear to affect the retention of irrelevant information. Memory loss for irrelevant associations was similar in the SLEEP and WAKE group, suggesting that retention of these associations was not differentially affected by sleep and wake. These results are in line with findings from a recent directed-forgetting study by Saletin and colleagues [8]. There, the retention of items that were cued to be remembered benefited from sleep, whereas memory for items that were cued to be forgotten showed no facilitation or impairment with sleep compared to a delay period without sleep.

We cannot completely exclude time-of-day effects with our experimental design. Although no design could remove circadian confounds during the encoding, delay, and retrieval phases without introducing sleep deprivation, a factor well known for its massive effect on many neural and endocrine systems [25], [26], it is possible that the effects reported here are in part due to the timing of our experiment. Regardless, the differences in memory performance between the relevant and the irrelevant category within the SLEEP or the WAKE group cannot be explained by any time of day effects. Moreover, the correlation between sleep time and retention for the relevant category in the SLEEP group provides additional support for a sleep-dependent effect of relevance on memory consolidation and helps minimizing concerns about circadian and/or interference interpretations of the key findings reported here. Furthermore, it should be noted that in daily life, circadian influences likely contribute to the mnemonic effects of sleep and as such could have served a similar purpose here [27].

In summary, we show for the first time that it is possible to modulate the retention of selected declarative memories after learning with simple verbal instructions on their future relevance. The finding that this effect depends on sleep demonstrates this intriguing state’s active role in memory consolidation and suggests that post-learning instruction can both help and hinder long-term retention.

## Materials and Methods

### Ethics Statement

The experiment was approved by the local medical ethical committee (CMO region Arnhem/Nijmegen) and was conducted in accordance with national legislation for the protection of human volunteers in non-clinical research settings and the Helsinki Declaration. Participants provided written informed consent before participating in this study.

### Participants

60 healthy participants (17 males; 7 left-handed; mean age: 22.1; age range 18–33) took part in the experiment. 10 participants were excluded from the analyses because they reported doubts or suspicions about the relevance instruction at debriefing. Therefore, 50 participants were included in the analyses reported in this study. All participants reported to be free of neurological or psychiatric illness and had normal or corrected-to-normal vision. Participants were paid or received course credits for participation. The specific reimbursement participants received consisted of a flat fee plus an additional sum based on their performance at the delayed retest.

### General Procedures

The experiment consisted of two sessions separated by a delay of 14 hours. During the first session, participants were briefed on the general procedures of the experiment, learned a collection of picture-location associations, were tested on their memory for these associations during the TEST, and were given a false instruction (see “Instruction” below) about the procedures of a second (identical) memory test, the RETEST. Participants returned to the lab for the second session after a 14 hour delay. Throughout the delay actigraphs recorded the activity of each participant. During the second session, participants were given the actual instruction for the RETEST, were tested once more on their memory for all associations and received a debriefing.

For half of the participants, the first session occurred in the morning, with RETEST at night, and no sleep in between (the “WAKE group”). For the other half, the first session occurred in the late afternoon, with RETEST in the morning, and the delay included a normal night of sleep (the “SLEEP group”).

### The Picture-Location Task

Participants learned 120 picture-location associations during the first session. Stimuli consisted of 60 color pictures of buildings and 60 color pictures of furniture items, all of the same size. Learning was conducted in 3 encoding-retrieval cycles; for an overview of the task, see also Figure 2.

During encoding, participants passively viewed each picture for 4s while it was associated with one of six possible locations on a computer screen, which was indicated by a color change of the associated location and the picture moving to that location. Presentation of building and furniture stimuli was randomly intermixed, but each picture category had its own set of six locations to limit the formation of across-category associations (i.e. linking particular furniture items to buildings at the same location as a mnemonic strategy). Additionally, the location array for each condition provided an implicit context specific to the picture categories that was encoded along with the associations.

Immediately after each encoding block, a retrieval block followed in which the participants were instructed to indicate the correct location of each picture. In this block, pictures were shown sequentially at the centre of the screen and participants chose the corresponding location for each picture with a joystick movement using their right hand. The picture stayed on screen for 4s; however, subjects were allowed to respond as soon as the picture appeared. In case the subject did not respond within the 4s, the picture went off screen but the trial lasted until the participant had made a response. No feedback was given. After a 1s interval, subjects were additionally instructed to rate the confidence of their response (1 =  unsure to 5 =  sure). In case they did not remember the location, they were instructed to indicate the lowest confidence rating of 1.

Each location was used equally often across the stimuli. Presentation of buildings and furniture stimuli was intermixed during both encoding and retrieval, and each phase used different random presentation orders for all stimuli.

The encoding–retrieval cycle was repeated three times with all individual pictures associated with one fixed location. The performance during the third retrieval phase was used as the baseline (“TEST”) memory performance score. After the 14 hour delay, participants returned and underwent one more retrieval phase without further encoding (“RETEST”).

### Instruction

Three sets of instructions were given during the course of the experiment. First, before the informed consent was signed, participants were instructed about the picture-location task and general procedures of the experiment. Second, a standard written and verbal relevance instruction was given to each participant by the experimenter after the TEST (i.e. when the learning phase and baseline test of the picture-location task had been completed). The instruction explained that only one picture category (furniture or buildings) would be tested at the RETEST, stressed the importance of the “relevant” category, and informed the participant about the added monetary bonus given for each correctly recalled relevant association at the RETEST. Assignment of buildings or furniture as the relevant picture category occurred in a counterbalanced manner across participants in both groups. The third set of instructions was given post-delay to inform participants on the procedure at RETEST. Specifically, when participants returned to the lab after the delay, they were re-instructed against their expectations that they would be tested on both picture categories, and that the monetary bonus would be given for all correct responses, regardless of category. Participants were asked during the debriefing at the end of the experiment whether they had any doubts or suspicions about the instructions given in this experiment. If participants expressed any doubts about the relevance instruction at any time during the experiment, they were excluded from the analyses reported in this article. 10 participants were excluded for this reason: 4 from the WAKE group (1 male, 1 left-handed) and 6 from the SLEEP group (1 male, all right-handed).

### Actigraphy and the Delay Period

After the relevance instruction was given, participants left the lab. Participants in the WAKE group were instructed not to nap but otherwise follow their normal daily routine. Participants from the SLEEP group were instructed to sleep normally and keep to habitual bedtimes. Throughout the delay period, each participant was monitored using wrist-mounted actigraphy (ActiGraph, Pensacola, USA). For all participants, activity logs were checked for the presence of sleep periods. The Total Sleep Time (in minutes) was subsequently calculated for each participant in the SLEEP group with the Cole-Kripke algorithm [28] as implemented in ActiLife 5 (Actigraph) and was subsequently used in the calculations of the correlations between sleep time and memory retention.
